# Crystal structure and Hirshfeld surface analysis of (*E*)-3-(2-chloro­phen­yl)-1-(2,5-di­chloro­thio­phen-3-yl)prop-2-en-1-one

**DOI:** 10.1107/S2056989018018066

**Published:** 2019-01-04

**Authors:** T. N. Sanjeeva Murthy, Zeliha Atioğlu, Mehmet Akkurt, M. K. Veeraiah, Ching Kheng Quah, C. S. Chidan Kumar, B. P. Siddaraju

**Affiliations:** a Department of Chemistry, Sri Siddhartha Academy of Higher Education, Tumkur 572 107, Karnataka, India; b İlke Education and Health Foundation, Cappadocia University, Cappadocia Vocational College, The Medical Imaging Techniques Program, 50420 Mustafapaşa, Ürgüp, Nevşehir, Turkey; c Department of Physics, Faculty of Sciences, Erciyes University, 38039 Kayseri, Turkey; d Department of Chemistry, Sri Siddhartha Institute of Technology, Tumkur 572 105, Karnataka, India; e X-ray Crystallography Unit, School of Physics, Universiti Sains Malaysia, 11800 USM, Penang, Malaysia; f Department of Engineering Chemistry, Vidya Vikas Institute of Engineering & Technology, Visvesvaraya Technological University, Alanahalli, Mysuru 570028, Karnataka, India; g Department of Chemistry, Cauvery Institute of Technology, Mandya 571 402, Karnataka, India

**Keywords:** crystal structure, 2,5- di­chloro­thio­phene ring, 2-chloro­phenyl ring, *E* configuration, Hirshfeld surface analysis, crystal structure

## Abstract

The mol­ecular structure of the title compound, C_13_H_7_Cl_3_OS, consists of a 2,5- di­chloro­thio­phene ring and a 2-chloro­phenyl ring linked *via* a prop-2-en-1-one spacer. The dihedral angle between the 2,5-di­chloro­thio­phene and 2-chloro­phenyl rings is 9.69 (12)°. The mol­ecule has an *E* configuration about the C=C bond and the carbonyl group is *syn* with respect to the C=C bond. The mol­ecular conformation is stabilized by two intra­molecular C—H⋯Cl contacts and one intra­molecular C—H⋯O contact, forming *S*(5)*S*(5)*S*(6) ring motifs. In the crystal, the mol­ecules are linked along the *a*-axis direction through van der Waals forces and along the *b* axis by face-to-face π-stacking between the thio­phene rings and between the benzene rings of neighbouring mol­ecules, forming corrugated sheets lying parallel to the *bc* plane. The inter­molecular inter­actions in the crystal packing were further analysed using Hirshfield surface analysis, which indicates that the most significant contacts are Cl⋯H/ H⋯Cl (28.6%), followed by C⋯H/H⋯C (11.9%), C⋯C (11.1%), H⋯H (11.0%), Cl⋯Cl (8.1%), O⋯H/H⋯O (8.0%) and S⋯H/H⋯S (6.6%).

## Abstract 2

The mol­ecular structure of the title compound consists of a 2,5-di­chloro­thio­phene ring and a 2-chloro­phenyl ring linked *via* a prop-2-en-1-one spacer. The mol­ecule has an *E* configuration about the C=C bond and the carbonyl group is *syn* with respect to the C=C bond. In the crystal, the mol­ecules are linked along the *a*-axis direction through van der Waals forces and by face-to-face π-stacking between the thio­phene rings and between the benzene rings of neighbouring mol­ecules along the *b* axis into zigzag sheets lying parallel to the *bc* plane.

## Chemical context   

Chalcone is an aromatic ketone that forms a central core for a variety of biological compounds, which are collectively known as chalcones. Chalcones, considered to be the precursors of flavonoids and isoflavonoids, are abundant in edible plants. They consist of open-chain flavonoids in which the two aromatic rings are joined by a three-carbon α,β-unsaturated carbonyl system. Chalcone was first isolated from Chinese liquorice (Glycyrrhizae inflata) (Rao *et al.*, 2004 ▸). It has a 1,3-diaryl-1-one skeletal system, which was recognized as the main pharmacophore for chalcones. The introduction of various substituents into the two aryl rings is also an area of inter­est for investigating structure–activity relationships. Chalcones are coloured compounds because of the presence of the –CO—CH=CH– chromophore. Different methods have been reported for the preparation of chalcones, the most convenient method being the Claisen-Schimdt condensation of equimolar qu­anti­ties of an aryl ­methyl­ketone with an aryl aldehyde in the presence of alcoholic alkali. The synthesis and anti­microbial evaluation of new chalcones containing a 2,5-di­chloro­thio­phene moiety have been reported (Tomar *et al.*, 2007 ▸). Recently, chalcones have been used in the field of materials science as non-linear optical devices (Raghavendra *et al.*, 2017 ▸; Chandra Shekhara Shetty *et al.*, 2016 ▸). The crystal structures of (*E*)-1-(2,5-di­chloro-3-thien­yl)-3-[4-(di­methyl­amino)­phen­yl]prop-2-en-1-one (Dutkiewicz *et al.*, 2010 ▸), (2*E*)-1-(2,5-di­chloro-3-thien­yl)-3-(6-meth­oxy-2-naphth­yl)prop-2-en-1-one (Jasinski *et al.*, 2010 ▸), (*E*)-1-(2,5-di­chloro-3-thien­yl)-3-(3,4-di­meth­oxy­phen­yl)prop-2-en-1-one (Harrison *et al.*, 2010*a* ▸), (*E*)-3-(2-chloro-4-fluoro­phen­yl)-1-(2,5-di­chloro­thio­phen-3-yl)prop-2-en-1-one (Sanjeeva Murthy *et al.*, 2018 ▸) and (2*E*)-3-(2,4-di­chloro­phen­yl)-1-(2,5-di­chloro­thio­phen-3-yl)prop-2-en-1-one (Murthy *et al.*, 2018 ▸) have previously been reported.
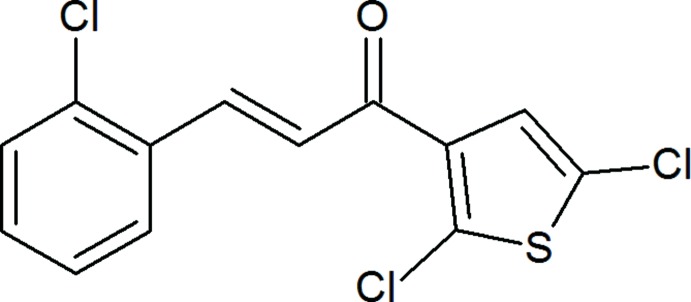


As part of our studies in this area, we report the crystal and mol­ecular structures of the title compound.

## Structural commentary   

**Figure 1:**
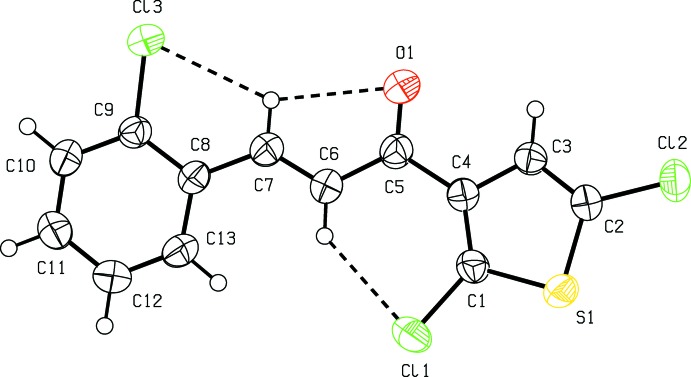
The mol­ecular structure of the title compound, showing the atom labelling and displacement ellipsoids drawn at the 50% probability level. Intra­molecular hydrogen bonds (Table 1 ▸) are shown as dashed lines.

As shown in Fig. 1 ▸, the title compound is constructed from two aromatic rings (2,5-di­chloro­thio­phene and terminal 2-chloro­phenyl rings), which are linked by a C=C—C(=O)—C enone bridge. The C3—C4—C5—O1 and O1—C5—C6—C7 torsion angles about the enone bridge are 6.7 (4) and 4.3 (4)°, respectively, probably as a result of steric repulsion between the chlorine atoms of adjacent mol­ecules. The dihedral angle between the 2-chloro­thio­phene and 2,4-di­chloro­phenyl rings is 9.69 (12)°. The bond lengths and angles in the title compound are comparable with those in the related compounds (2*E*)-3-(2,4-di­chloro­phen­yl)-1-(2,5-di­chloro­thio­phen-3-yl)prop-2-en-1-one (Sanjeeva Murthy *et al.*, 2018 ▸), (*E*)-3-(3,4-di­meth­oxy­phen­yl)-1-(1-hy­droxy­naphthalen-2­yl)prop-2-en-1-one (Ezhilarasi *et al.*, 2015 ▸), (*E*)-1-(3-bromo­phen­yl)-3-(3,4-di­meth­oxy­phen­yl)-prop-2-en-1-one (Escobar *et al.*, 2012 ▸) and (*E*)-3-(2-bromo­phen­yl)-1-(3,4-di­meth­oxy­phen­yl)prop-2-en-1-one (Li *et al.*, 2012 ▸). The mol­ecular conformation is stabilized by two intra­molecular C—H⋯Cl contacts and one intra­molecular C—H⋯O contact (Table 1 ▸), forming *S*(5)*S*(5)*S*(6) ring motifs.

## Supra­molecular features   

**Figure 2:**
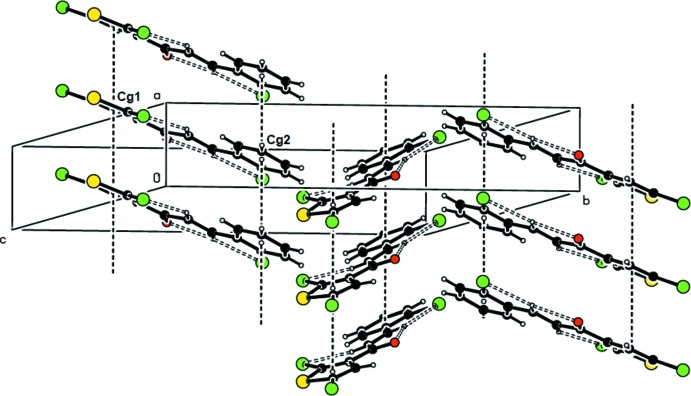
View along the *b*-axis direction of the zigzag sheets lying parallel to (011). π-stacking is observed between the thio­phene rings (centroid *Cg*1) of adjacent molecules in alternating sheets along the [100] direction and between the benzene rings (centroid *Cg*2) of the same molecules.

In the crystal, conventional hydrogen bonds are not observed. Mol­ecules are linked along the *a*-axis direction through van der Waals forces. π-stacking is observed between thio­phene rings (S1/C1–C4, centroid *Cg*1) of adjacent mol­ecules in alternating sheets along the [100] direction [*Cg*1⋯*Cg*1^i,ii^: centroid–centroid distance = 3.902 (2) Å, shortest perpendic­ular distance for the centroid of one ring to the plane of the other = 3.597 (1) Å, ring-centroid offset = 1.512 Å; symmetry codes: (i) −1 + *x*, *y*, *z*; (ii) 1 + *x*, *y*, *z*] and between the benzene rings (C8–C13, centroid *Cg*2) of the same mol­ecules [*Cg*2⋯*Cg*2^i,ii^: centroid–centroid distance = 3.902 (2) Å, shortest perpendicular distance = 3.482 (1) Å, offset = 1.760 Å]. The mol­ecules are packed into corrugated sheets lying parallel to (011) (Figs. 2 ▸ and 3 ▸). Details of Cl⋯H and O⋯H contacts are given in Table 2 ▸.

## Hirshfeld surface analysis   

**Figure 4:**
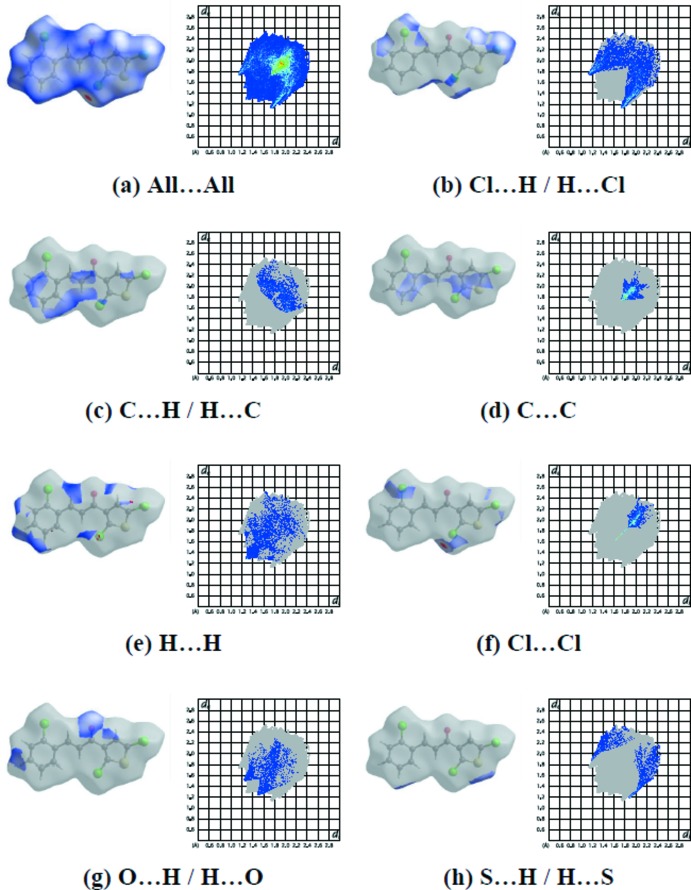
The two-dimensional fingerprint plots for the title compound, showing (*a*) all inter­actions, and delineated into (*b*) Cl⋯H/H⋯Cl, (*c*) C⋯H/H⋯C, (*d*) C⋯C, (*e*) H⋯H, (*f*) Cl⋯Cl, (*g*) O⋯H/H⋯O and (*h*) S⋯H/H⋯S inter­actions.

Hirshfeld surfaces and fingerprint plots were generated for the title compound using *CrystalExplorer* (McKinnon *et al.*, 2007 ▸). Hirshfeld surfaces enable the visualization of inter­molecular inter­actions by using different colours and colour intensity to represent short or long contacts and indicate the relative strength of the inter­actions. The overall two-dimensional fingerprint plot for the title compound and those delineated into Cl⋯H/H⋯Cl, C⋯H/H⋯C, C⋯C, H⋯H, Cl⋯Cl, O⋯H/H⋯O and S⋯H/H⋯S contacts are illustrated in Fig. 4 ▸; the percentage contributions from the different inter­atomic contacts to the Hirshfeld surfaces are as follows: Cl⋯H/H⋯Cl (28.6%), C⋯H/H⋯C (11.9%), C⋯C (11.1%), H⋯H (11.0%), Cl⋯Cl (8.1%), O⋯H/H⋯O (8.0%) and S⋯H/H⋯S (6.6%). The contributions of the other weak inter­molecular contacts to the Hirshfeld surfaces are listed in Table 3 ▸.

The C—H⋯Cl inter­actions appear as two distinct spikes in the fingerprint plot [Fig. 4 ▸(*b*)] with *d*_e_ + *d*_i_ ≃ 2.85 Å [*d*_e_ and *d*_i_ represent the distances from a point on the Hirshfeld surface to the nearest atoms outside (external) and inside (inter­nal) the surface, respectively]. The C⋯H/H⋯C inter­actions are shown in Fig. 4 ▸(*c*). The scattered points show the van der Waals contacts and π–π stacking inter­actions. The inter­atomic C⋯C contacts appear as an arrow-shaped distribution of points in Fig. 4 ▸(*d*), with the vertex at *d*_e_ = *d*_i_ = 1.75 Å. The C⋯C contacts reflect π–π inter­actions between the aromatic rings. The H⋯H inter­actions are reflected in Fig. 4 ▸(*e*) as widely scattered points of high density due to the large hydrogen content of the mol­ecule. The split spike with the tip at *d*_e_ = *d*_i_ ≃ 1.3 Å is due to the short inter­atomic H⋯H contacts. Cl⋯Cl contacts [Fig. 4 ▸(*f*)] are disfavoured when the number of H atoms on the mol­ecular surface is large because of competition with the more attractive H⋯Cl contacts. Cl⋯Cl contacts from a parallel alignment of C—Cl bonds [C1—Cl1⋯Cl1^iii^, and C2—Cl2⋯C10^iv^; symmetry codes: (iii) 2 − *x*, 1 − *y*, 1 − *z*; (iv) 2 − *x*, 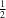 + *y*, 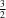 − *z*] may be indicated. They are known in the literature as type-I halogen–halogen inter­actions (Bui *et al.*, 2009 ▸), with both C—Cl⋯Cl angles equal to one another. In the present case, these angles are close to 165°. The H⋯O/O⋯H contacts [Fig. 4 ▸(*g*)] also have a symmetrical distribution of points, with two pairs of thin and thick edges at *d*_e_ + *d*_i_ ≃ 2.75 Å. The S⋯H contacts shown in Fig. 4 ▸(*h*) are contracted to a much lesser degree.

The large number of Cl⋯H/H⋯Cl, C⋯H/H⋯C, C⋯C, H⋯H, Cl⋯Cl, O⋯H/H⋯O and S⋯H/H⋯S inter­actions suggest that van der Waals inter­actions and hydrogen bonding play the major roles in the crystal packing (Hathwar *et al.*, 2015 ▸).

## Database survey   

The closest related compounds with the same skeleton and containing a similar bis-chalcone moiety to the title compound but with different substituents on the aromatic rings are: (2*E*)- 1-(5-chloro­thio­phen-2-yl)-3-(4-ethyl­phen­yl)prop-2-en-1-one [(I); Naik *et al.*, 2015 ▸], (2*E*)-1-(5-bromo­thio­phen-2-yl)-3-(4-ethyl­phen­yl)prop- 2-en-1-one [(II); Naik *et al.*, 2015 ▸], (2*E*)-1-(5-chloro­thio­phen-2-yl)-3-(4-eth­oxy­phen­yl)prop-2-en-1-one [(III); Naik *et al.*, 2015 ▸], (2*E*)-1-(5-bromo­thio­phen-2-yl)-3-(4- eth­oxy­phen­yl)prop-2-en-1-one [(IV); Naik *et al.*, 2015 ▸], (2*E*)- 3-(4-bromo­phen­yl)-1-(5-chloro­thio­phen-2-yl)prop-2-en-1-one [(V); Naik *et al.*, 2015 ▸], (2*E*)-1-(5-bromo­thio­phen-2-yl)-3-(3- meth­oxy­phen­yl)prop-2-en-1-one [(VI); Naik *et al.*, 2015 ▸], (*E*)- 1-(5-chloro­thio­phen-2-yl)-3-(*p*-tol­yl)prop-2-en-1-one [(VII); Kumara *et al.*, 2017 ▸], (*E*)-1-(5-chloro­thio­phen-2-yl)-3-(2,4-di­methyl­phen­yl) prop-2-en-1-one [(VIII); Naveen *et al.*, 2016 ▸], (2*E*)-1-(5-bromo­thio­phen- 2-yl)-3-(2-chloro­phen­yl)prop-2-en- 1-one [(IX); Anitha *et al.*, 2015 ▸], (2*E*)-1-[4-hy­droxy-3- (morpholin-4-ylmeth­yl)phen­yl]-3-(thio­phen-2-yl)prop-2-en-1- one [(X); Yesilyurt *et al.*, 2018 ▸], (*E*)-1-(2-amino­phen­yl)-3- (thio­phen-2-yl)prop-2-en-1-one [(XI); Chantrapromma *et al.*, 2013 ▸] and (2E)-3-(2,4-di­chloro­phen­yl)-1-(2,5-di­chloro­thio­phen- 3-yl)prop-2-en-1-one [(XII); Sanjeeva Murthy *et al.*, 2018 ▸]. In (I) and (II), the structures are isostructural in space group *P*1, while (III) and (IV) are isostructural in space group *P*2_1_/*c*. There are no hydrogen bonds of any kind in the structures of compounds (I) and (II), but in the structures of compounds (III) and (IV), the mol­ecules are linked into *C*(7) chains by means of C—H⋯O hydrogen bonds. In (V), there are again no hydrogen bonds nor any π–π stacking inter­actions but in (VI), the mol­ecules are linked into *C*(5) chains by C—H⋯O hydrogen bonds. In each of compounds (I)–(VI), the mol­ecular skeletons are close to planarity, and there are short halogen–halogen contacts in the structures of compounds (II) and (V) and a short Br⋯O contact in the structure of compound (VI). In (VII), the mol­ecule is non-planar, with a dihedral angle of 22.6 (2)° between the aromatic rings. The mol­ecules are linked by pairs of C—H⋯π inter­actions, forming inversion dimers. There are no other significant inter­molecular inter­actions present. In (VIII), the mol­ecule is nearly planar, the dihedral angle between the thio­phene and phenyl rings being 9.07 (8)°. The mol­ecules are linked via weak C—H⋯O and C—H⋯S hydrogen bonds, forming chains propagating along the *c*-axis direction. In (IX), the thienyl ring is not coplanar with the benzene ring, their planes forming a dihedral angle of 13.2 (4)°. In the crystal, mol­ecules stack along the *a*-axis direction, with the inter­planar separation between the thienyl rings and between the benzene rings being 3.925 (6) Å. In (X), the thio­phene ring forms a dihedral angle of 26.04 (9)° with the benzene ring. The mol­ecular conformation is stabilized by an O—H⋯N hydrogen bond. The mol­ecules are connected through C—H⋯O hydrogen bonds, forming wave-like layers parallel to the *ab* plane, which are further linked into a three-dimensional network by C—H⋯π inter­actions. In (XI), the mol­ecule is almost planar with a dihedral angle of 3.73 (8)° between the phenyl and thio­phene rings. An intra­molecular N—H⋯O hydrogen bond generates an *S*(6) ring motif. Adjacent mol­ecules are linked into dimers in an anti-parallel face-to-face manner by pairs of C—H⋯O inter­actions. Neighbouring dimers are further linked into chains along the *c*-axis direction by N—H⋯N hydrogen bonds. In (XII), the dihedral angle between the thio­phene and benzene rings increases to12.24 (15)°. The mol­ecular conformation is stabilized by intra­molecular C—H⋯Cl contacts, forming *S*(6) and *S*(5) ring motifs. In the crystal, the mol­ecules are linked through face-to-face π-stacking between the thio­phene rings and the benzene rings of the mol­ecules into zigzag sheets lying parallel to the *bc* plane.

## Synthesis and crystallization   

The title compound was synthesized by a reported procedure (Kumar *et al.*, 2013*a* ▸,*b* ▸). 1-(2,5-Di­chloro­thio­phen-3-yl)ethan­one (0.01 mol) (Harrison *et al.*, 2010*b* ▸) and 2-chloro­benzaldehyde (0.01 mol) were dissolved in 20 ml of methanol. A catalytic amount of NaOH was added to the solution dropwise with vigorous stirring. The reaction mixture was stirred for about 4 h at room temperature. The formed crude products were filtered, washed successively with distilled water and recrystallized from methanol. The melting point (352–363 K) was determined using a Stuart Scientific (UK) apparatus.

## Refinement   

Crystal data, data collection and structure refinement details are summarized in Table 4 ▸. H atoms were positioned geometrically and refined using riding model, with C—H = 0.93–0.96 Å and *U*_iso_(H) = 1.2*U*_eq_(C) or 1.5*U*_eq_(C-meth­yl).

## Supplementary Material

Crystal structure: contains datablock(s) I. DOI: 10.1107/S2056989018018066/qm2131sup1.cif

Structure factors: contains datablock(s) I. DOI: 10.1107/S2056989018018066/qm2131Isup2.hkl

Supporting information file. DOI: 10.1107/S2056989018018066/qm2131Isup3.cml

CCDC reference: 1036796

Additional supporting information:  crystallographic information; 3D view; checkCIF report

## Figures and Tables

**Figure 3:**
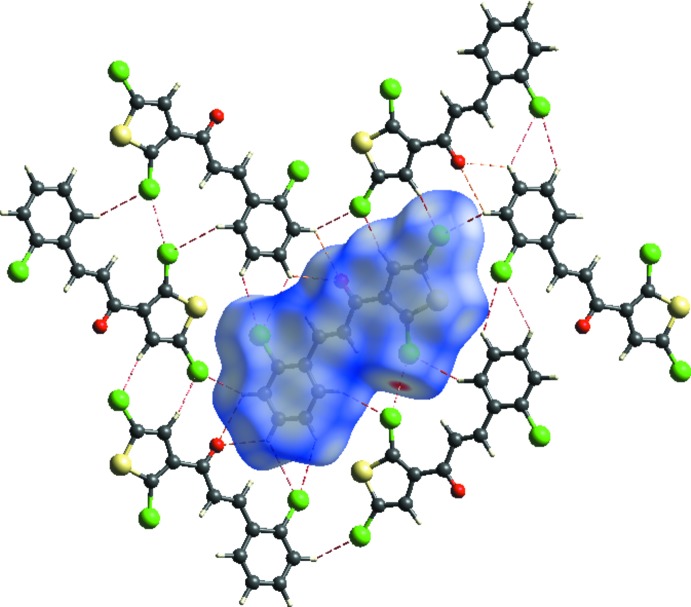
Hirshfeld surface mapped *d*_norm_ showing the intra- and inter­molecular C—H⋯Cl and C—H⋯O hydrogen-bonded contacts.

**Table 1:** Hydrogen-bond geometry (Å, °)

*D*—H⋯*A*	*D*—H	H⋯*A*	*D*⋯*A*	*D*—H⋯*A*
C6—H6*A*⋯Cl1	0.93	2.54	3.245 (3)	133
C7—H7*A*⋯Cl3	0.93	2.59	3.043 (3)	110
C7—H7*A*⋯O1	0.93	2.46	2.790 (3)	101

**Table 2:** Summary of short inter­atomic contacts (Å) in the title compound

Contact	distance	Symmetry operation
Cl1⋯Cl1	3.2876 (11)	2 − *x*, 1 − *y*, 1 − *z*
Cl2⋯C10	3.618 (3)	2 − *x*, 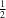 + *y*, 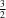 − *z*
Cl2⋯H10*A*	3.06	1 − *x*, 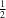 + *y*, 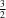 − *z*
H3*A*⋯Cl2	3.01	2 − *x*, 1 − *y*, 2 − *z*
H3*A*⋯Cl2	2.98	3 − *x*, 1 − *y*, 2 − *z*
Cl3⋯H12*A*	3.11	*x*, 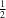 − *y*, 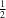 + *z*
C6⋯C7	3.504 (4)	1 + *x*, *y*, *z*
O1⋯H10*A*	2.85	1 + *x*, 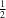 − *y*, 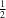 + *z*

**Table 3:** Percentage contributions of inter­atomic contacts to the Hirshfeld surface for the compound

Contact	Percentage contribution
Cl⋯H/H⋯Cl	28.6
C⋯H/H⋯C	11.9
C⋯C	11.1
H⋯H	11.0
Cl⋯Cl	8.1
O⋯H/H⋯O	8.0
S⋯H/H⋯S	6.6
C⋯Cl/Cl⋯C	4.7
S⋯Cl/Cl⋯S	4.1
S⋯C/C⋯S	2.1
O⋯C/C⋯O	1.6
O⋯Cl/Cl⋯O	1.0
S⋯S	0.8
O⋯O	0.3

**Table 4:** Experimental details

Crystal data	
Chemical formula	C_13_H_7_Cl_3_OS
*M*_r_	317.60
Crystal system, space group	Monoclinic, *P*2_1_/*c*
Temperature (K)	294
*a*, *b*, *c* (Å)	3.9017 (6), 22.038 (3), 15.127 (2)
β (°)	96.998 (3)
*V* (Å^3^)	1291.0 (3)
*Z*	4
Radiation type	Mo *K*α
μ (mm^−1^)	0.85
Crystal size (mm)	0.56 × 0.10 × 0.06

Data collection	
Diffractometer	Bruker APEXII CCD
Absorption correction	Multi-scan (*SADABS*; Sheldrick, 2007 ▸)
*T*_min_, *T*_max_	0.907, 0.953
No. of measured, independent and observed [*I* > 2σ(*I*)] reflections	10683, 2669, 1977
*R*_int_	0.039
(sin θ/λ)_max_ (Å^−1^)	0.630

Refinement	
*R*[*F* ^2^ > 2σ(*F* ^2^)], *wR*(*F* ^2^), *S*	0.038, 0.099, 1.08
No. of reflections	2669
No. of parameters	163
H-atom treatment	H-atom parameters constrained
Δρ_max_, Δρ_min_ (e Å^−3^)	0.30, −0.32

Computer programs: *APEX2* and *SAINT* (Bruker, 2007 ▸), *SHELXS97* (Sheldrick, 2008 ▸), *SHELXL2014* (Sheldrick, 2015 ▸), *ORTEP-3 for Windows* (Farrugia, 2012 ▸) and *PLATON* (Spek, 2009 ▸).

## supplementary crystallographic information

### Crystal data

**Table:**

C_13_H_7_Cl_3_OS	*F*(000) = 640
*M**_r_* = 317.60	*D*_x_ = 1.634 Mg m^−^^3^
Monoclinic, *P*2_1_/*c*	Mo *K*α radiation, λ = 0.71073 Å
*a* = 3.9017 (6) Å	Cell parameters from 2207 reflections
*b* = 22.038 (3) Å	θ = 2.7–23.3°
*c* = 15.127 (2) Å	µ = 0.85 mm^−^^1^
β = 96.998 (3)°	*T* = 294 K
*V* = 1291.0 (3) Å^3^	Needle, colourless
*Z* = 4	0.56 × 0.10 × 0.06 mm

### Data collection

**Table:**

Bruker APEXII CCD diffractometer	1977 reflections with *I* > 2σ(*I*)
φ and ω scans	*R*_int_ = 0.039
Absorption correction: multi-scan (SADABS; Sheldrick, 2007)	θ_max_ = 26.6°, θ_min_ = 1.6°
*T*_min_ = 0.907, *T*_max_ = 0.953	*h* = −4→4
10683 measured reflections	*k* = −25→27
2669 independent reflections	*l* = −18→18

### Refinement

**Table:**

Refinement on *F*^2^	0 restraints
Least-squares matrix: full	Hydrogen site location: inferred from neighbouring sites
*R*[*F*^2^ > 2σ(*F*^2^)] = 0.038	H-atom parameters constrained
*wR*(*F*^2^) = 0.099	*w* = 1/[σ^2^(*F*_o_^2^) + (0.0397*P*)^2^ + 0.3125*P*] where *P* = (*F*_o_^2^ + 2*F*_c_^2^)/3
*S* = 1.08	(Δ/σ)_max_ = 0.001
2669 reflections	Δρ_max_ = 0.30 e Å^−^^3^
163 parameters	Δρ_min_ = −0.32 e Å^−^^3^

### Special details

**Table:**

Geometry. All esds (except the esd in the dihedral angle between two l.s. planes) are estimated using the full covariance matrix. The cell esds are taken into account individually in the estimation of esds in distances, angles and torsion angles; correlations between esds in cell parameters are only used when they are defined by crystal symmetry. An approximate (isotropic) treatment of cell esds is used for estimating esds involving l.s. planes.

### Fractional atomic coordinates and isotropic or equivalent isotropic displacement parameters (Å^2^)

**Table:**

	*x*	*y*	*z*	*U*_iso_*/*U*_eq_	
S1	1.19136 (18)	0.57814 (3)	0.76052 (4)	0.0475 (2)	
O1	0.7421 (6)	0.37898 (9)	0.81944 (13)	0.0729 (7)	
Cl1	0.8918 (3)	0.51901 (4)	0.59796 (4)	0.0767 (3)	
Cl2	1.37875 (18)	0.58175 (3)	0.95615 (4)	0.0553 (2)	
Cl3	0.18192 (19)	0.21002 (3)	0.65287 (4)	0.0547 (2)	
C1	1.0021 (6)	0.51465 (11)	0.71070 (15)	0.0419 (6)	
C2	1.2074 (6)	0.54379 (11)	0.86299 (15)	0.0405 (6)	
C3	1.0742 (6)	0.48785 (11)	0.85791 (16)	0.0409 (6)	
H3A	1.062770	0.463252	0.907420	0.049*	
C4	0.9511 (6)	0.46945 (11)	0.76907 (15)	0.0368 (5)	
C5	0.7905 (7)	0.40836 (12)	0.75452 (17)	0.0432 (6)	
C6	0.6952 (7)	0.38479 (12)	0.66419 (17)	0.0498 (7)	
H6A	0.750113	0.407394	0.615933	0.060*	
C7	0.5354 (7)	0.33292 (12)	0.64948 (16)	0.0442 (6)	
H7A	0.484449	0.311865	0.699544	0.053*	
C8	0.4278 (6)	0.30426 (11)	0.56358 (16)	0.0383 (5)	
C9	0.2658 (6)	0.24794 (11)	0.55730 (16)	0.0389 (6)	
C10	0.1648 (7)	0.22027 (12)	0.47628 (17)	0.0478 (6)	
H10A	0.058424	0.182449	0.474158	0.057*	
C11	0.2228 (8)	0.24897 (13)	0.39919 (17)	0.0548 (7)	
H11A	0.153881	0.230816	0.344393	0.066*	
C12	0.3828 (8)	0.30463 (13)	0.40288 (18)	0.0568 (7)	
H12A	0.423175	0.324019	0.350527	0.068*	
C13	0.4827 (7)	0.33156 (12)	0.48330 (17)	0.0503 (7)	
H13A	0.590560	0.369228	0.484531	0.060*	

### Atomic displacement parameters (Å^2^)

**Table:**

	*U*^11^	*U*^22^	*U*^33^	*U*^12^	*U*^13^	*U*^23^
S1	0.0609 (4)	0.0364 (4)	0.0465 (4)	−0.0055 (3)	0.0117 (3)	−0.0010 (3)
O1	0.1185 (19)	0.0477 (12)	0.0502 (11)	−0.0279 (12)	0.0013 (12)	0.0062 (10)
Cl1	0.1343 (8)	0.0581 (5)	0.0363 (4)	−0.0125 (5)	0.0043 (4)	0.0020 (3)
Cl2	0.0611 (4)	0.0495 (4)	0.0514 (4)	−0.0053 (3)	−0.0085 (3)	−0.0104 (3)
Cl3	0.0722 (5)	0.0451 (4)	0.0472 (4)	−0.0141 (3)	0.0086 (3)	0.0062 (3)
C1	0.0527 (15)	0.0377 (15)	0.0357 (12)	0.0016 (12)	0.0069 (11)	−0.0030 (10)
C2	0.0395 (13)	0.0417 (15)	0.0395 (13)	0.0005 (11)	0.0013 (10)	−0.0045 (11)
C3	0.0456 (14)	0.0375 (14)	0.0382 (12)	−0.0001 (11)	0.0001 (11)	0.0019 (11)
C4	0.0366 (12)	0.0349 (14)	0.0387 (12)	0.0033 (10)	0.0029 (10)	−0.0009 (10)
C5	0.0476 (14)	0.0356 (14)	0.0452 (14)	0.0002 (11)	0.0010 (11)	0.0000 (11)
C6	0.0636 (17)	0.0416 (16)	0.0434 (14)	−0.0111 (13)	0.0028 (12)	−0.0013 (12)
C7	0.0496 (15)	0.0376 (15)	0.0448 (13)	−0.0028 (12)	0.0031 (11)	0.0013 (11)
C8	0.0373 (13)	0.0332 (14)	0.0440 (13)	−0.0011 (10)	0.0037 (10)	0.0006 (10)
C9	0.0414 (13)	0.0340 (14)	0.0412 (13)	0.0004 (11)	0.0042 (10)	0.0023 (10)
C10	0.0518 (15)	0.0399 (15)	0.0508 (15)	−0.0081 (12)	0.0023 (12)	−0.0030 (12)
C11	0.0685 (19)	0.0525 (18)	0.0419 (14)	−0.0038 (15)	0.0011 (13)	−0.0041 (13)
C12	0.0710 (19)	0.0553 (19)	0.0434 (15)	−0.0100 (15)	0.0043 (13)	0.0090 (13)
C13	0.0586 (17)	0.0383 (15)	0.0532 (15)	−0.0082 (13)	0.0041 (13)	0.0068 (12)

### Geometric parameters (Å, º)

**Table:**

S1—C1	1.713 (3)	C6—H6A	0.9300
S1—C2	1.719 (2)	C7—C8	1.460 (3)
O1—C5	1.210 (3)	C7—H7A	0.9300
Cl1—C1	1.710 (2)	C8—C9	1.391 (3)
Cl2—C2	1.704 (2)	C8—C13	1.395 (3)
Cl3—C9	1.735 (2)	C9—C10	1.382 (3)
C1—C4	1.362 (3)	C10—C11	1.369 (4)
C2—C3	1.336 (3)	C10—H10A	0.9300
C3—C4	1.430 (3)	C11—C12	1.374 (4)
C3—H3A	0.9300	C11—H11A	0.9300
C4—C5	1.490 (3)	C12—C13	1.367 (4)
C5—C6	1.467 (3)	C12—H12A	0.9300
C6—C7	1.308 (3)	C13—H13A	0.9300
			
C1—S1—C2	90.19 (12)	C6—C7—H7A	116.2
C4—C1—Cl1	130.5 (2)	C8—C7—H7A	116.2
C4—C1—S1	113.67 (18)	C9—C8—C13	116.2 (2)
Cl1—C1—S1	115.81 (14)	C9—C8—C7	121.7 (2)
C3—C2—Cl2	127.67 (19)	C13—C8—C7	122.0 (2)
C3—C2—S1	112.53 (18)	C10—C9—C8	122.2 (2)
Cl2—C2—S1	119.80 (15)	C10—C9—Cl3	117.64 (19)
C2—C3—C4	113.5 (2)	C8—C9—Cl3	120.21 (18)
C2—C3—H3A	123.2	C11—C10—C9	119.5 (2)
C4—C3—H3A	123.2	C11—C10—H10A	120.2
C1—C4—C3	110.1 (2)	C9—C10—H10A	120.2
C1—C4—C5	131.1 (2)	C10—C11—C12	119.9 (2)
C3—C4—C5	118.8 (2)	C10—C11—H11A	120.0
O1—C5—C6	121.3 (2)	C12—C11—H11A	120.0
O1—C5—C4	117.9 (2)	C13—C12—C11	120.2 (3)
C6—C5—C4	120.8 (2)	C13—C12—H12A	119.9
C7—C6—C5	122.0 (2)	C11—C12—H12A	119.9
C7—C6—H6A	119.0	C12—C13—C8	122.0 (2)
C5—C6—H6A	119.0	C12—C13—H13A	119.0
C6—C7—C8	127.5 (2)	C8—C13—H13A	119.0
			
C2—S1—C1—C4	0.4 (2)	O1—C5—C6—C7	4.3 (4)
C2—S1—C1—Cl1	177.73 (16)	C4—C5—C6—C7	−175.9 (2)
C1—S1—C2—C3	−0.3 (2)	C5—C6—C7—C8	−179.7 (2)
C1—S1—C2—Cl2	179.98 (16)	C6—C7—C8—C9	178.5 (3)
Cl2—C2—C3—C4	179.88 (19)	C6—C7—C8—C13	−1.2 (4)
S1—C2—C3—C4	0.2 (3)	C13—C8—C9—C10	0.1 (4)
Cl1—C1—C4—C3	−177.2 (2)	C7—C8—C9—C10	−179.6 (2)
S1—C1—C4—C3	−0.3 (3)	C13—C8—C9—Cl3	−179.60 (19)
Cl1—C1—C4—C5	1.6 (4)	C7—C8—C9—Cl3	0.7 (3)
S1—C1—C4—C5	178.5 (2)	C8—C9—C10—C11	−0.4 (4)
C2—C3—C4—C1	0.0 (3)	Cl3—C9—C10—C11	179.3 (2)
C2—C3—C4—C5	−178.9 (2)	C9—C10—C11—C12	0.5 (4)
C1—C4—C5—O1	−172.0 (3)	C10—C11—C12—C13	−0.3 (5)
C3—C4—C5—O1	6.7 (4)	C11—C12—C13—C8	0.0 (5)
C1—C4—C5—C6	8.1 (4)	C9—C8—C13—C12	0.1 (4)
C3—C4—C5—C6	−173.2 (2)	C7—C8—C13—C12	179.8 (3)

### Hydrogen-bond geometry (Å, º)

**Table:**

*D*—H···*A*	*D*—H	H···*A*	*D*···*A*	*D*—H···*A*
C6—H6*A*···Cl1	0.93	2.54	3.245 (3)	133
C7—H7*A*···Cl3	0.93	2.59	3.043 (3)	110
C7—H7*A*···O1	0.93	2.46	2.790 (3)	101
